# Utilization of fine needle aspiration cytology at Kamuzu central hospital

**DOI:** 10.1371/journal.pone.0196561

**Published:** 2018-06-12

**Authors:** Shiraz Khan, George Liomba, Nora E. Rosenberg, Christopher Stanley, Cocxilly Kampani, Bal Mukunda Dhungel, Mina C. Hosseinipour

**Affiliations:** 1 University of North Carolina Project, Lilongwe, Malawi; 2 Kamuzu Central Hospital, Lilongwe, Malawi; 3 University of North Carolina, Chapel Hill, North Carolina, United States of America; Universita degli Studi di Torino, ITALY

## Abstract

**Background:**

Fine needle aspiration cytology (FNAC) has been widely accepted to be a safe, accurate, prompt and inexpensive procedure for diagnosis of both neoplastic and infectious diseases in adult and pediatric populations. Despite its value for diagnosis, FNAC is underutilized in resource limited countries. We reviewed the utilization of FNAC after it was introduced at Kamuzu Central Hospital (KCH).

**Methods:**

A retrospective review of all FNAC performed at KCH laboratory during the period of January 2012 to July 2014 was conducted using an electronic database from KCH laboratory. We evaluated factors associated with a diagnostic sample using multivariate logistic regression model.

**Results:**

750 FNAC were reviewed from 722 patients: 56.9% were adults >15 years and 54% were female. The number of FNAC increased annually from 56 (2012) to 379 (2013) to 315 (up to July 2014). Of 750 FNAC, 56.4% were performed by non-pathologists. The most common sites were lymph nodes (38.1%), abdomen (25.8%), breast (16.3%), and head & neck (15.7%). Most of the samples (77.6%) were diagnostic. FNAC was more likely to be diagnostic if performed by pathologists versus non-pathologists (OR 1.78, 95% CI 1.20–2.64), in 2013 compared to 2012 (OR 1.95, 95% CI 1.05–3.56), or performed on a deep lesion versus a subcutaneous lesion (OR 1.71, 95% CI 1.15–2.5), or if samples were taken from the head and neck (OR 2.4, 95% CI: 1.39–4.39), and abdomen (OR 2.66, 95%CI1.59–4.42) compared to those from the lymph nodes. The odds of a diagnostic test did not differ significantly according to gender, HIV status, or age groups.

**Conclusion:**

Most FNACs successfully diagnosed the presence or absence of disease, with substantial improvements over time. However, training for non-pathologists may facilitate more diagnostic results.

## Introduction

In resource-limited countries (RLC), including Malawi, pathology services are scarce resulting in significant challenges in clinical management[1,2]. Without pathology services, medical practitioners have diagnostic barriers and are forced to make clinical judgments to manage a variety of diseases[3,4]. This situation may result in providing incorrect and potentially harmful treatments as well as misuse of the limited resources. For example, lymphadenopathy maybe treated as tuberculosis, when the true diagnosis is a malignancy, thereby delaying effective treatment[5].

Efforts have been made in RLC to develop and establish pathology centers to help curb this problem. In 2011, a collaboration between Malawi Ministry of Health (MoH) and University of North Carolina (UNC) resulted in the establishment of a pathology laboratory at KCH[6]. The pathology laboratory currently provides histopathology, cytology including FNAC (Fine Needle Aspiration Cytology), immunohistochemistry studies, and maintains an active telepathology program with UNC Pathologists[7].

FNAC has been used as a pathologic diagnostic procedure for over 60 years and has been proven to be an inexpensive, prompt, accurate and less technically demanding way of obtaining a specimen for diagnosis[8–12].Due to its simplicity, low complication rate, cost effectiveness and high patient acceptance, FNAC is a valuable tool in RLC where other diagnostic modalities are limited. FNAC provides a definitive tissue diagnosis to fill in the gap for many diagnostic dilemmas[8–12] especially when used in conjunction with cell block and IHC studies. In some countries, FNAC has been approved as the first line diagnostic tool for palpable superficial lesions[13].

Despite the beneficial attributes of FNAC, the procedure is still underutilized in RLC[14].Clinicians understanding of the benefits of FNAC and acquiring skills to perform FNAC may encourage its wide spread use in these settings.

At KCH, FNAC was introduced in 2012. We characterized the FNAC specimens, including their distribution as per the anatomical sites, turn-around times, and whether they were diagnostic or non-diagnostic and described the factors associated with diagnostic FNACs.

## Methodology

This is a retrospective review of all FNACs performed at KCH during a period from January 2012 to July 2014. KCH is a 1,250 bedded referral hospital for central Malawi and serves a catchment area of approximately about 5 million people.

### Data collection and management

The study population included both children and adults undergoing FNAC procedure at KCH. Data was collected using a standardized cytology requisition form and were keyed in a Microsoft Access electronic database.

The information collected which was included in this analysis were; age, sex, HIV & ART status, anatomical site, clinical diagnosis and cytological diagnosis, and date of collection. The performer of FNAC procedure was not indicated on the form. However, the day of the week was calculated from the collection dates and used as a proxy to see who performed the FNAC procedure (pathologist or non-pathologist). The pathologist has an outpatient FNAC clinic on tuesdays and other clinicians usually performed FNAC on the other days. Non pathologists included all clinicians. Radiologist did not perform FNAC and only guided with ultrasound when pathologists attempted FNAC of difficult to reach deep lesions. FNAC sites were classified according to the anatomical region: head and neck (excluding cervical lymph nodes), lymph nodes, abdomen, breast, chest &back and extremities. Lesions were also classified as subcutaneous or deep, depending on their proximity to the skin. All lesions apart from skin and subcutaneous lesions were considered deep seated, thus all abdominal and most ultrasound guided FNAC were deep seated except for some breast and thyroid masses.

The results were classified to be either diagnostic or non-diagnostic. Diagnostic referred to those results with sufficient material for interpretation. Those that were diagnostic were further classified as malignant, suspicious for malignancy, benign, tuberculosis (TB) infection and other infections. We classified the malignant lesions further to carcinoma, lymphoma, sarcoma, and unclassified, according to the cytology report. The diagnoses were reported by experienced Pathologists onsite and only diagnostically challenging cases and those cases enrolled for ongoing lymphoma research were only reviewed through tele-pathology with UNC Pathologist[7,15].

### Statistical analyses

Distribution of categorical baseline data have been presented using proportions and percentages, and compared between groups using Pearson Chi-squared tests. Continuous normally-distributed data have been presented as medians with inter-quartile ranges. Factors associated with whether a result was diagnostic were estimated with odds ratios (OR) and 95% confidence intervals (CI) using multivariate logistic models. In all analyses, statistical significance was considered at a 2-sided α-level of 0.05. All analyses were performed in Stata SE 12.1.

### Ethical consideration

The Malawi National Health Sciences Committee approved the study. The University of North Carolina Institutional review board also granted approval to this analysis. All analysis was conducted on de-identified data.

## Results

A total of 750 FNAC samples were analyzed from 722 patients at KCH pathology laboratory between January 2012 and July 2014. The median turnaround time of the FNAC results from sample acquisition to final report was 3 (IQR 1–6) days.

The time for processing of the samples once received in the laboratory to final report was 2 days (IQR1-4).

**Fig 1:**
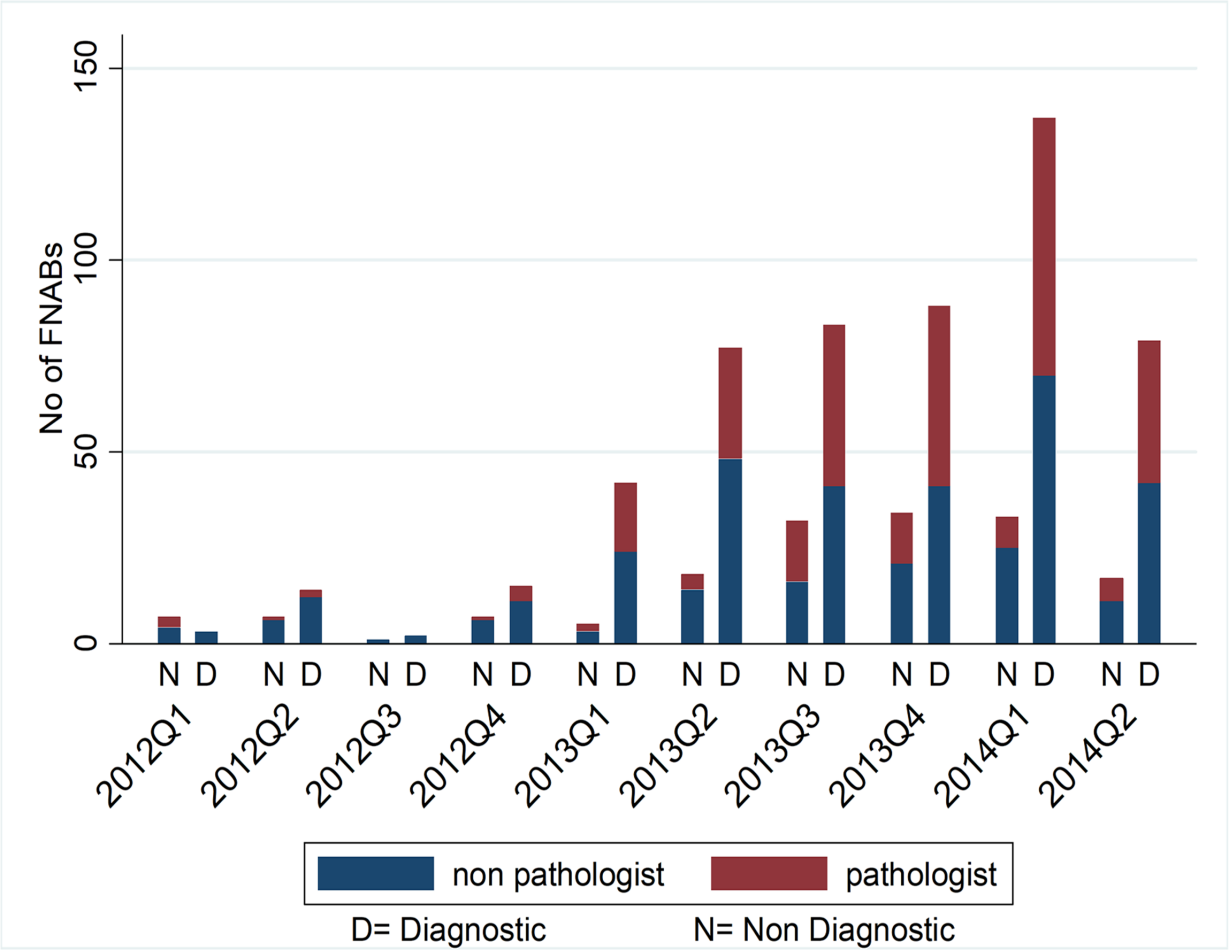
Number of FNAs done by pathologists and non-pathologists over time.

The number of FNACs increased annually from 56 in 2012 to 379 in 2013 and to 315 in the first half of 2014 (Fig 1). Of these samples, 56.9% (n = 427) were adults and 43.1% (n = 323) were pediatric. Four hundred and three (54%) of the samples were from females, 342 (46%) were from males, and five had a missing value. (Table 1). Non-pathologists performed 56.4% (n = 423) and pathologists performed 43.6% (n = 327). The primary distribution of sites was as follows: lymph nodes 38.1% (n = 286), abdomen 25.7% (n = 193), breast 16.3% (n = 122), and head & neck 15.7% (n = 118). Other sites included thoracolumbar region 2.0% (n = 15) and extremities 2.1% (n = 16).

**Table 1:** Baseline characteristics of FNA samples.

Variable	Total n = 750 (%)	Pediatric n = 324	Adult (n = 427)
**Gender**			
Female	403 (54.1)	146 (45.9)	257 (60.2)
Male	342 (45.9)	172 (54.1)	170 (39.8)
**HIV status**			
Negative	392(52.3)	261 (80.8)	131(30.7)
Positive	160(21.3)	7 (2.2)	153(35.8)
Unknown	198(26.4)	55 (17)	143(33.5)
**Aspirator**			
Pathologist	327 (43.6)	76(23.5)	251(58.8)
Non-Pathologist	423 (56.4)	247(76.5)	176(41.2)
**Aspirated Sites**			
Lymph nodes	286 (38.1)	109(33.8)	177(41.5)
Abdomen	193 (25.7)	128(39.6)	65(15.2)
Breast	122(16.3)	7(2.2)	115(2.7)
Head &Neck	118(15.7)	64(19.8)	54(7.5)
Thoracolumbar region	15(2.1)	7(1.9)	8(2)
Extremities	16(2.1)	8(2.5)	8(1.9)
**Diagnosis**			
Diagnostic	582 (77.6)	249 (77.2)	333 (78.0)
Non Diagnostic	168 (22.4)	74 (22.8)	94 (22.0)
**Diagnosis Classified**			
Malignant	343(45.7)	183(56.8)	160(37.5)
Suspicious for Malignancy	45(6.0)	21(6.5)	24(5.6)
Benign Lesions	130(17.3)	23 (7.1)	107(25.1)
TB infection	32(4.3)	6 (1.9)	26(6.1
Other Infections	32(4.3)	16(4.9)	16(3.8)
**Malignant Lesions**			
Lymphoma	129(37.7)	105(57.4)	24(15.1)
Carcinoma	118(34.1)	7(3.8)	111(69.2)
Sarcoma	38(11.1)	25(13.7)	13(8.2)
Unclassified	58(17.1)	46(25.1)	12 (7.6)
**Median Turnaround Time- IQR (Days)**			
Sample Acquisition→Result	3(1–6)	4 (1–6)	2(1–7)
Laboratory →Result	2(1–4)	2 (1–5)	2(1–4)

Overall, 77.6% (n = 582) of the samples were diagnostic. Amongst the diagnostic samples, the most common diagnosis was malignant lesions, 58.9% (n = 343) followed by benign lesions 22.3% (n = 130), suspicious for malignancy 7.7% (n = 45), tuberculosis (TB) 5.5% (n = 32) and other infections 5.5% (n = 32). Of the 582, 92.3% had a definitive diagnosis and no further testing was required. From the malignant lesions, lymphoma was the most common diagnosis (37.7%, n = 129) followed by carcinoma (34.2%, n = 118), unclassified (17%, n = 58) and sarcoma (11.1%, n = 38). Of the non-diagnostic samples (n = 168), half of them were from lymph nodes, followed by abdomen, breast, head & neck, chest, back, and extremities (Table 1).

Obtaining a diagnostic test did not differ by gender, HIV status, or age group. When a FNAC was performed by a pathologist, a diagnostic yield was more likely than if it was performed by a non-pathologist (OR 1.93, 95% CI 1.27–2.94). Specifically, 67% of non-diagnostic results were for the FNACs performed by the non-pathologists.

An FNAC was more likely to be diagnostic when performed in 2014 than 2012 (OR 2.3, 95% CI 1.24–4.35). When a FNAC was performed on a deep seated lesions, a diagnostic yield was more likely than for the subcutaneous lesions (OR 1.71, 95% CI 1.15–2.5). As compared to FNACs from lymph nodes, a diagnostic yield was more likely for the FNACs performed from the abdomen (OR = 2.66, 95% CI 1.59–4.42) or the head & neck (2.4, 95% CI 1.39–4.39). However, there was no differences with lesions from the breast, thoracolumbar region and extremities compared to the lymph nodes. (Table 2)

**Table 2:** ODDS ratios for factors associated with a diagnostic FNAC.

	Unadjusted		Adjusted	
Variable	OR	(95% CI)	OR	(95% CI)
**Gender**	** **			
Female	1		1	
Male	0.89	0.63–1.26	0.97	0.63–1.29
**Age group**	** **			
<15	1		1	
≥15	1.05	0.75–1.49	0.91	0.59–1.41
**HIV status**	** **			
Negative	1		1	
Positive	0.93	0.60–1.45	0.96	0.56–1.65
Unknown	0.96	0.63–1.44	0.85	0.54–1.34
**Performed by Pathologist**				
No	1		1	
Yes	1.74	1.22–2.49	1.78	1.20–2.64
**Tumor Location**				
Superficial	1		1	
Deep	1.66	1.16–2.38	1.71	1.15–2.5
**Period**				
1Jan12-31Dec12	1		1	
1Jan13-31Dec13	2.11	1.17–3.79	1.95	1.05–3.56
1Jan 14-31Jul14	2.93	1.59–5.38	2.67	1.53–5.05
**Site of Lesion**				
Lymph node	1		1	
Abdomen	2.35	1.48–3.76	2.66	1.59–4.42
Breast	1.54	0.93–2.54	1.67	0.93–3.02
Head &Neck	2.04	1.18–3.51	2.4	1.39–4.39
Thoracolumbar region & Extremities	1.02	0.45–2.31	1.29	0.55–3.01

## Discussion

For the first time, we describe the experiences and lessons learned from the introduction of FNAC in a resource constraint referral hospital in Malawi. After FNAC was introduced, the utilization of the procedure and the diagnostic accuracy increased over time, and the majority of the results were available within 72 hours, demonstrating feasibility and improvement over time. FNACs were more diagnostic when the aspirator was a pathologist as compared to other clinicians (Non-pathologist). Deep seated lesions had more diagnostic yields than superficial lesions. Diagnostic yield for lesions of the head & neck and abdomen lesions was higher compared to lymph nodes. These observations suggest that there is still room for improvement.

FNAC has been known to have a diagnostic accuracy as high as 97% [16]and has been recommended as a first line diagnostic tool for palpable masses[17]. In our study, 77.6% of the samples had a diagnostic outcome and, of these, 92.3% had a definitive diagnosis, where a standard diagnosis was assigned for definitive therapy available at KCH. Some cases were followed up with biopsy confirmation and in a few cases we did immunohistochemistry to confirm further. Most of the malignant lesions were lymphoma (37.7%, n = 129) and this result could be partially attributed to the lymphoma research study trails going on in our hospital. Having a reliable diagnostic test is vital as it means patients get timely and appropriate care at a lower costs, as the need for repeat FNAC or tissue biopsy is minimized[18]. Additionally, this bedside test prevents overburdening minor operating theaters and surgical staff that might be required for more invasive open biopsies and surgeries.

Our analysis showed that FNAC was more diagnostic when performed by a pathologist, compared to other clinicians. This finding is consistent with various studies[14,16,18,19] that have shown that the success of FNAC is largely dependent on the training and experience of the individual performing the procedure. There is generally more diagnostic accuracy when conducted by an experienced pathologist.

After the introduction of FNAC at KCH, the utilization of the procedure increased over time, suggesting increased comfort, acceptance, and awareness among clinicians. Furthermore, the FNAC became more diagnostic over time (Fig 1). This finding could be related to the aspirator, as pathologists did more aspirations over time and also the possibility that non-pathologist aspirators showed improvement with more exposure when they did more FNACs.

In the Malawian setting where there are only 3 pathologists for a 16 million population, trained clinicians capable of performing the FNAC procedure will increase the utilization and improve the specimen adequacy. Study has shown that clinicians with formal training in FNAC only had 2% non-diagnostic samples as compared to 25% of clinicians without formal training [16]. In another study conducted in Blantyre Malawi, similar outcomes were observed when the procedure was done by a trained nurse versus a pathologist on patients with Burkitt’s lymphoma[14].

When compared to superficial lesions, deep seated lesions were more diagnostic. This finding is surprising since superficial lesions should be easier to aspirate and hence should have more diagnostic yield. In our setting, clinicians tend to refer patients with deep lesions due to fear of complications and also a feeling that they will not be successful on these cases. FNACs from deep seated lesions also included ultrasound guided FNACs, thereby increasing the accuracy. Considering these factors, the samples from deep-seated lesions were more likely to be aspirated by a pathologist and under ultrasound guidance, thereby providing a plausible reason as to why FNACs from deep lesions were more diagnostic in our study. However, despite pathologists performing more of the aspiration from deep lesions it was not significantly different from non-pathologist.

When compared to lymph nodes, FNACs from the abdomen and head & neck were more diagnostic however there was no difference with the FNACs from the breast or thoracolumbar lesions. This finding also concurs with the possibility of referral to a pathologist as sites from the abdomen and head and neck are more likely to be referred to the pathologists and many of them were performed under ultrasound or CT scan guidance.

FNAC results were generally available within 3 days of procedure. In few cases longer turnaround time was related to delays in transportation of the sample to the laboratory as the processing and interpretation of the samples occurred within 2 days.

Our analysis included all of the samples for period of 2 years and seven months and were therefore representative of those samples collected at this facility. These observations were conducted in a resource limited hospital, and thus results may be similar in other high-volume resource-limited settings.

We note several limitations in our study. Our evaluation was limited, as it did not contain information on additional important variables that might influence results (e.g. needle size and training of non-pathologist aspirators). The type of needle used was not controlled in this review hence the possibility that inexperienced clinicians may have used a smaller or large bore needle for FNA which could have contributed to some samples being non diagnostic. Similarly, we were unable to ascertain the training level of the non-pathologists performing the FNACs, as those with better training in other settings have been observed to have a higher diagnostic yield[18].The day of week was used as a proxy for the type of person who performed the FNAC, and this may have resulted in misclassification of some of the FNA procedures in terms of the aspirator. Lastly, our review did not estimate how accurate the FNAC results were, as histopathology and cytology correlation study could not be performed as the results could not be easily traced due to lack of record of the pathology numbers on the clinical requisitions forms and most patients were not followed up. We plan to conduct a future study where the accuracy of FNACs in our setting shall be estimated and some of these limitations could be addressed.

## Conclusion

This study further supports that FNAC has promising diagnostic potential and its introduction in a resource limited tertiary hospital, proved very useful to diagnose many lesions that would have been previously missed.

Our study reaffirms the fact that FNAC can be easily introduced and implemented in resource limited settings and we encourage the roll out of this procedure to many tertiary care hospitals in other RLC.

## Supporting information

S1 File fna_data final.xls- Plos.xls. (XLS)

S2 File FNAs dataset_edited.dta. (DTA)

## References

[1] Adesina A, Chumba D, Nelson AM, Orem J, Roberts DJ, Wabinga H, et al Improvement of pathology in sub-Saharan Africa. Lancet Oncol [Internet]. 2013;14(4):e152–7. Available from: http://dx.doi.org/10.1016/S1470-2045(12)70598-3 doi: 10.1016/S1470-2045(12)70598-3 23561746 10.1016/S1470-2045(12)70598-3

[2] Harder HI. Pathology services in developing countries. Arch Pathol Lab Med. 2009;133(December):1911 doi: 10.1043/1543-2165-133.12.1911 19961243 10.5858/133.12.1911

[3] Hay Burgess DC, Wasserman J, Dahl C a. Global health diagnostics. Nature. 2006;444 Suppl(December):1–2. 10.1038/nature05440 17159888

[4] Girosi F, Olmsted SS, Keeler E, Hay Burgess DC, Lim Y-W, Aledort JE, et al Developing and interpreting models to improve diagnostics in developing countries. Nature. 2006;444 Suppl:3–8. doi: 10.1038/nature05441 17159889 10.1038/nature05441

[5] Mabedi C, Kendig C, Liomba G, Shores C, Chimzimu F, Kampani C, et al Causes of cervical lymphadenopathy at kamuzu central hospital. Malawi Med J. 2014;26(1):16–9. 24959320 PMC4062779

[6] Gopal S, Krysiak R, Liomba NG, Horner MJ, Shores CG, Alide N, et al Early Experience after Developing a Pathology Laboratory in Malawi, with Emphasis on Cancer Diagnoses. PLoS One. 2013;8(8):6–13. 10.1371/journal.pone.0070361 PMC3737192 23950924

[7] Montgomery ND, Liomba NG, Kampani C, Krysiak R, Stanley CC, Tomoka T, et al Accurate real-time diagnosis of lymphoproliferative disorders in Malawi through clinicopathologic teleconferences: A model for pathology services in sub-Saharan Africa. Am J Clin Pathol. 2016;146(4):423–30. doi: 10.1093/ajcp/aqw118 27594430 10.1093/ajcp/aqw118 PMC5040876

[8] Das DK. Fine-needle aspiration cytology: Its origin, development, and present status with special reference to a developing country, India. Diagn Cytopathol. 2003;28(6):345–51. doi: 10.1002/dc.10289 12768643 10.1002/dc.10289

[9] Razack R, Michelow P, Leiman G, Harnekar A, Poole J, Wessels G, et al An interinstitutional review of the value of FNAB in pediatric oncology in resource-limited countries. Diagn Cytopathol. 2012;40(9):770–6. doi: 10.1002/dc.21624 22888083 10.1002/dc.21624

[10] Polyzos SA, Anastasilakis AD. Systematic review of cases reporting blood extravasation-related complications after thyroid fine-needle biopsy. J Otolaryngol—Head Neck Surg. 2010;39(5):532–41. 20828516

[11] Agarwal D, Khare A, Ansari M. Evaluation of fine needle aspiration biopsy as a diagnostic tool in pediatric head and neck lesions. Pathol Lab Med Int [Internet]. 2010;2:131–6. Available from: http://www.dovepress.com/getfile.php?fileID=8185&origin=publication_detail

[12] Monaco SE, Teot LA. Cytopathology of pediatric malignancies. Cancer Cytopathol. 2014;122(5):322–36. doi: 10.1002/cncy.21401 24604906 10.1002/cncy.21401

[13] Patel MM, Patel K, Kaptan K. Original Article Fine Needle Aspiration Cytology As a First Line. Natl J Med Res. 2013;3(2):106–10.

[14] Wright CA, Pienaar JP, Marais BJ. Fine needle aspiration biopsy: diagnostic utility in resource-limited settings. Ann Trop Paediatr [Internet]. 2008;28(1):65–70. Available from: http://www.tandfonline.com/doi/full/10.1179/146532808X270707 doi: 10.1179/146532808X270707 18318952 10.1179/146532808X270707

[15] Montgomery ND, Tomoka T, Krysiak R, Powers E, Mulenga M, Kampani C, et al Practical Successes in Telepathology Experiences in Africa. Clin Lab Med. 2017;(2017). 10.1016/j.cll.2017.10.011 PMC5996143 29412878

[16] Ljung BM, Drejet A, Chiampi N, Jeffrey J, Goodson WH, Chew K, et al Diagnostic accuracy of fine-needle aspiration biopsy is determined by physician training in sampling technique. Cancer. 2001;93(4):263–8. 11507700 10.1002/cncr.9040

[17] Florentine BD, Staymates B, Rabadi M, Barstis J, Black A. The reliability of fine-needle aspiration biopsy as the initial diagnostic procedure for palpable masses: A 4-year experience of 730 patients from a community hospital-based outpatient aspiration biopsy clinic. Cancer. 2006;107(2):406–16. doi: 10.1002/cncr.21976 16773630 10.1002/cncr.21976

[18] Etit D, Tugyan N, Avci A, Altinel D, Tan A, Arslanoglu S. An evaluation of nondiagnostic fine needle aspiration biopsy results: The importance of having an experienced cytopathologist. Turkish J Med Sci. 2011;41 (4)(4):609–13.

[19] Wu M, Burstein DE, Yuan S, Nurse L a, Szporn AH, Zhang D, et al A comparative study of 200 fine needle aspiration biopsies performed by clinicians and cytopathologists. Laryngoscope [Internet]. 2006;116(7):1212–5. Available from: http://www.ncbi.nlm.nih.gov/pubmed/16826062 doi: 10.1097/01.mlg.0000224507.07560.28 16826062 10.1097/01.mlg.0000224507.07560.28

